# Editorial: Technological innovations and pancreatic cancer

**DOI:** 10.3389/fonc.2024.1497367

**Published:** 2024-10-07

**Authors:** Mikolaj Kowal, Andrew Smith, Sanjay Pandanaboyana, Samir Pathak

**Affiliations:** ^1^ The Department of Hepatobiliary Surgery, St. James’s University Hospital, Leeds, United Kingdom; ^2^ Leeds Institute of Medical Research, Faculty of Medicine and Health, University of Leeds, Leeds, United Kingdom; ^3^ Hepato-Pancreato-Biliary and Transplant Unit, Freeman Hospital, Newcastle Upon Tyne, United Kingdom

**Keywords:** pancreas - adenocarcinoma, pancreas, pancrea ticoduodenectomy, technology, innovation

**Figure 1:**
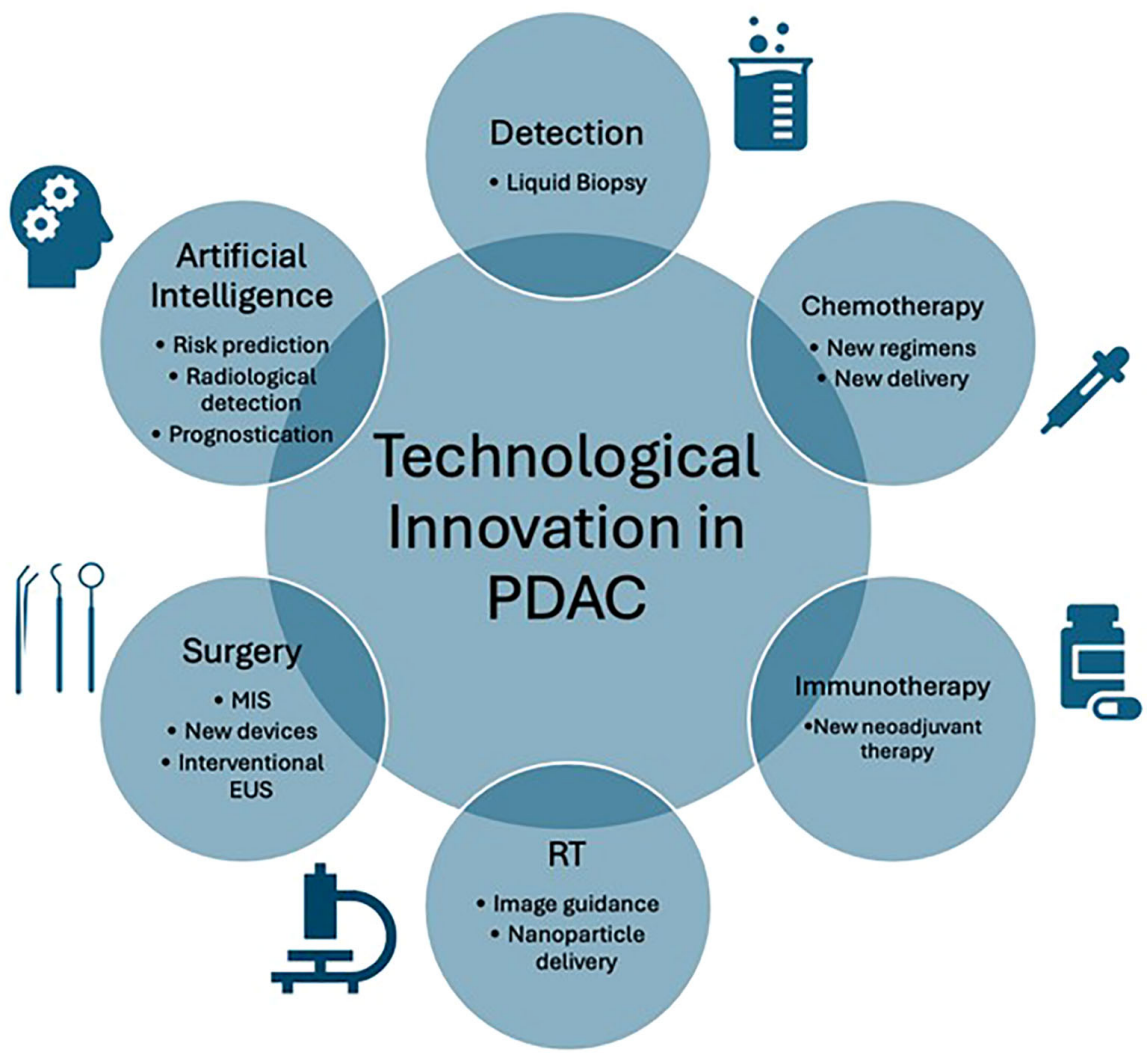
Graphical representation of technological innovation in pancreatic ductal adenocarcinoma (PDAC). EUS, Endoscopic ultrasound; MIS, Minimally invasive surgery; RT, Radiotherapy.

Pancreatic ductal adenocarcinoma (PDAC) has poor survival outcomes. The main reasons include late presentation, resulting in only 20% of patients being eligible for surgery, and poor response to chemotherapy, secondary to the challenging tumour biology (1, 2). For patients undergoing surgery, the local anatomy and invasiveness of PDAC results in high operative risks, with morbidity and mortality estimated at 50-70% and 2-8%, respectively (3, 4). The incidence of PDAC is rising, making it the fourth leading cause of death in the United States (5). It is imperative to prioritise PDAC research. Technological advances may improve patient outcomes through early detection and optimisation of treatments. The editorial highlights the latest technological advances in PDAC and identifies areas for research (**Figure 1**).

Technological breakthroughs enabling early detection of PDAC were described in a review on liquid biopsy (6). This sampling method uses biomarkers such as circulating tumour DNA (ctDNA) and extracellular vesicles (EVs). Liquid biopsy is exciting due to its non-invasive, real-time and repeatable properties. Recent advances in methylation analysis, detecting epigenetic reprogramming in early tumorigenesis, have improved the detection of PDAC using ctDNA. A meta-analysis performed in 2019 showed sensitivity and specificity rates of 70% and 86% respectively. Another biomarker showing promise are EVs, with one study showing high sensitivity and specificity rates of 99% and 82% respectively. The current accuracy of liquid biopsy across all methods for PDAC was investigated in a meta-analysis (7). They concluded that liquid biopsy using biomarkers such as ctDNA was less accurate than CA19-9 for PDAC detection. However, ctDNA was associated with worse survival, making it a useful prognostication tool. Further research utilising multiple biomarkers may increase the accuracy in PDAC detection.

Advances in neoadjuvant chemotherapy regimens are enabling surgical treatment for patients with locally advanced PDAC. Zhang et al. documented the case of a patient with a locally advanced PDAC. Following six cycles of GEM-NabP chemotherapy the patient underwent surgery, with histology confirming near-complete response. Successes such as this are often attributed to favourable biology, but standardisation of neoadjuvant regimens may improve overall outcomes. Further improvements in neoadjuvant treatments are seen in combination with immunotherapy. Lu et al. published a case report of combined neoadjuvant treatment using a programmed cell death protein-1 inhibitor and chemoradiotherapy (8). Following neoadjuvant treatment for a locally advanced PDAC, the patient underwent a pylorus-preserving pancreaticoduodenectomy (PPPD) with histology showing a pathologic complete response. Further data is also showing the benefit of established adjuvant chemotherapy regimens. A retrospective cohort study by Choi et al. analysed outcomes for patients who received adjuvant chemotherapy for locally advanced PDAC. They showed that 5-fluorouracil-based regimens resulted in favourable survival. New advances have also been made in the delivery of chemotherapy. Cao et al. performed a meta-analysis of regional intra-arterial chemotherapy (RIAC) compared with systemic treatment. RIAC has been developed recently and trialled in PDAC due to its ability to deliver high concentrations locally, while maintaining low systemic drug levels. Their analysis concluded that patients who received RIAC had a higher rate of partial remission and fewer complications. The studies highlighted show progress within chemotherapy and immunotherapy. There is an urgent need for standardisation of regimens, combination therapy and drug delivery.

Technological advances in radiotherapy (RT) for PDAC were highlighted in a review article by Malla et al. (9). Despite the ability to convert borderline resectable cases to surgical resection, up to a third of patients can die during RT from disease progression. Advances in Stereotactic Body Radiation Therapy (SBRT) and hypofractionation enable the delivery of biologically effective doses with reduced toxicity to surrounding tissues. Specifically, innovation in magnetic resonance-guided on-table adaptive RT is enabling this. A recent phase two trial demonstrated reduced incidence of acute grade 3+ gastrointestinal toxicity at 90 days post treatment. New trials are also being conducted on nanoparticles to enhance RT delivery. Hafnium oxide nanoparticles NBTXR3 are activated by RT to improve radiation-induced abscopal effects. These technologies are likely to improve the effects of neoadjuvant RT for patients with PDAC. Healthcare professionals will have the tools to deliver highly effective doses of RT without the tissue toxicity that currently limits the treatment potential of this therapy.

For patients eligible for surgery, advances in surgical technology are improving outcomes. The uptake of minimally invasive surgery (MIS) for PDAC was analysed by Yan et al. in their meta-analysis of laparoscopic versus open PPPD (10). The 39 studies demonstrated reduced morbidity, length of stay (LOS), blood loss, delayed gastric emptying as well as higher R0 rates in laparoscopic PPPD. The authors importantly highlight that only four randomised controlled studies (RCTs) were included. Two of these were multi-centre and all non-randomised trials were retrospective, showing a need for standardised high-quality surgical trials. The advances of MIS have since been taken further with the advent of robotic surgery. The recently published RCT concerning PDAC has demonstrated safety of the robotic approach for PPPD (11). Novel surgical devices are also prospects for improving patient outcomes. Sheen et al. compared the AEON™ endovascular stapler with traditional devices for laparoscopic distal pancreatectomy in 58 patients (12). Their analysis of using AEON™, characterised by uniform staple lengths and a multi-firing gear, showed a reduction in post-operative day three drain lipase and postoperative pancreatic fistula (POPF) from 65% to 20%. Similar advances have been made in endoscopic ultrasound (EUS). On et al. described the utility of EUS in PDAC in their review. Innovation in the field has enabled EUS-guided interventions such biliary drainage, gastrojejunostomy, coeliac plexus blocks and fiducial placements. Further uptake of interventional EUS is currently limited by a paucity of prospective RCTs. New technologies should be evaluated according to standardised frameworks, such as Idea, Development, Exploration, Assessment and Long-term follow-up, to develop evidence of clinical benefit for patients (13).

One technological area which could improve all aspects of PDAC treatment is Artificial Intelligence (AI). Zhao et al. reviewed the latest achievements of AI in PDAC. Recent advances include AI-based radiomics which can detect PDAC on imaging, and deep learning which can produce precision models for risk prediction and prognostication in PDAC. AI has been used to produce three-dimensional models of tumours, enabling thorough operative planning and improving outcomes such as operative time, blood loss and LOS. One study used artificial neural network models for PDAC prognostication, outperforming corresponding logistic regression models in predicting survival (14).

In summary, technological innovation is transforming the landscape of PDAC treatment. Healthcare professionals are empowered with new tools for detection, therapy delivery, surgery and data analysis in PDAC. Further translational research is needed in multi-biomarker liquid biopsy and RCTs on standardisation of neoadjuvant/adjuvant therapies. Surgical technologies should be evaluated in a robust framework with high quality trials to confer effective and safe treatments for patients with PDAC.
